# Comparison of motion correction techniques applied to functional near-infrared spectroscopy data from children (Erratum)

**DOI:** 10.1117/1.JBO.25.5.059801

**Published:** 2020-05-12

**Authors:** Xiao-Su Hu, Maria M. Arredondo, Megan Gomba, Nicole Confer, Alexandre F. DaSilva, Timothy D. Johnson, Mark Shalinsky, Ioulia Kovelman

**Affiliations:** a University of Michigan, Center for Human Growth and Development, Ann Arbor, Michigan, United States; b University of Michigan, Department of Psychology, Ann Arbor, Michigan, United States; c University of Michigan, School of Dentistry, Headache and Orofacial Pain Effort Laboratory, Biologic and Materials Sciences Department, Ann Arbor, Michigan, United States; d University of Michigan, Department of Biostatistics, School of Public Health, Ann Arbor, Michigan, United States

## Abstract

The erratum concerns amendment of Section 2.5 of the published article.

This article [*J. Biomed. Opt.* **20**(12), 126003 (2015) doi: 10.1117/1.JBO.20.12.126003] was originally published online on 11 December 2015 with an erroneous Sec. 2.5, “Motion Artifacts Correction Methods.” Specifically, the authors belatedly noticed that Secs. 2.5.1–2.5.4 mistakenly included 25% overlap with portions of a previously published work, “Motion artifacts in functional near-infrared spectroscopy: a comparison of motion correction techniques applied to real cognitive data,” by S. Brigadoi et al. (PMCID: PMC3762942).

While the Methods section of the Hu et al 2015 article included text from a previously published article, the authors affirm that the investigation, data collection, and findings were both novel and comprised of previously unpublished content.

Section 2.5 has been amended, with multiple checks to ensure the originality and accuracy of the description while also acknowledging that the approach is modeled upon Brigadoi et al. (2014).

This article was corrected online on 28 April 2020.

